# Polymeric DNase-I nanozymes targeting neutrophil extracellular traps for the treatment of bowel inflammation

**DOI:** 10.1186/s40580-024-00414-9

**Published:** 2024-02-08

**Authors:** Chi-Pin James Wang, Ga Ryang Ko, Yun Young Lee, Juwon Park, Wooram Park, Tae-Eun Park, Yoonhee Jin, Se-Na Kim, Jung Seung Lee, Chun Gwon Park

**Affiliations:** 1 Department of Biomedical Engineering, SKKU Institute for Convergence, Sungkyunkwan University (SKKU), Suwon, Gyeonggi 16419 Republic of Korea; 2 Department of Intelligent Precision Healthcare Convergence, SKKU Institute for Convergence, Sungkyunkwan University (SKKU), Suwon, Gyeonggi 16419 Republic of Korea; 3 Department of Biomedical Engineering, College of Medicine, Seoul National University, Seoul, 03080 Republic of Korea; 4 Department of Tropical Medicine, Medical Microbiology, and Pharmacology, John A. Burns School of Medicine, University of Hawai’i at Manoa, Honolulu, HI 96813 USA; 5 Department of Integrative Biotechnology, College of Biotechnology and Bioengineering, Sungkyunkwan University (SKKU), Suwon, Gyeonggi 16419 Republic of Korea; 6 Department of Biomedical Engineering, Ulsan National Institute of Science and Technology (UNIST), Ulsan, 44919 Republic of Korea; 7 Department of Physiology, Yonsei University College of Medicine, Seoul, 03722 Republic of Korea; 8 Research and Development Center, MediArk Inc., Cheongju, Chungbuk 28644 Republic of Korea; 9 Department of Industrial Cosmetic Science, College of Bio-Health University System, Chungbuk National University, Cheongju, Chungbuk 28644 Republic of Korea; 10 Biomedical Institute for Convergence at SKKU (BICS), Sungkyunkwan University (SKKU), Suwon, Gyeonggi 16419 Republic of Korea

**Keywords:** Neutrophil extracellular trap, DNase-I, Nanoparticle, Colitis, Inflammatory bowel disease

## Abstract

Inflammatory bowel disease (IBD), including Crohn’s disease and ulcerative colitis, is a family of chronic disorders along the gastrointestinal tract. Because of its idiopathic nature, IBD does not have a fundamental cure; current available therapies for IBD are limited to prolonged doses of immunomodulatory agents. While these treatments may reduce inflammation, limited therapeutic efficacy, inconsistency across patients, and adverse side effects from aggressive medications remain as major drawbacks. Recently, excessive production and accumulation of neutrophil extracellular traps (NETs) also known as NETosis have been identified to exacerbate inflammatory responses and induce further tissue damage in IBD. Such discovery invited many researchers to investigate NETs as a potential therapeutic target. DNase-I is a natural agent that can effectively destroy NETs and, therefore, potentially reduce NETs-induced inflammations even without the use of aggressive drugs. However, low stability and rapid clearance of DNase-I remain as major limitations for further therapeutic applications. In this research, polymeric nanozymes were fabricated to increase the delivery and therapeutic efficacy of DNase-I. DNase-I was immobilized on the surface of polymeric nanoparticles to maintain its enzymatic properties while extending its activity in the colon. Delivery of DNase-I using this platform allowed enhanced stability and prolonged activity of DNase-I with minimal toxicity. When administered to animal models of IBD, DNase-I nanozymes successfully alleviated various pathophysiological symptoms of IBD. More importantly, DNase-I nanozyme administration successfully attenuated neutrophil infiltration and NETosis in the colon compared to free DNase-I or mesalamine.

**Supplementary Information:**

The online version contains supplementary material available at 10.1186/s40580-024-00414-9.

## Introduction

Inflammatory bowel disease (IBD), including Crohn’s disease and ulcerative colitis, is a family of chronic disorders along the gastrointestinal tract characterized by its idiopathic and relapsing nature [1]. Following the compounding increase in the global prevalence of IBD [2], multiple factors (e.g., environmental, microbial, ethnical, or genetic etc.) have been identified as potential mediators of IBD development [3]; however, the precise onset and pathogenesis of IBD remain largely obscure [4]. To date, the majority of therapeutic drugs used against IBD are immunosuppressive drugs, steroids, 5-aminosalicylates, or other agents intended to inhibit inflammation [5]. Given that IBD symptoms are highly relapsing and remitting, IBD patients frequently require long-term treatments to sustain remission. Furthermore, such prolonged administration of immunosuppressive drugs greatly increases the risks of drug-related side effects, toxicities, and secondary infections [6, 7]. To decrease the clinical burden associated with long-term IBD treatment, novel therapeutic approaches that could minimize the use of immunosuppressive agents are urgently required.

Neutrophil infiltration has been traditionally considered as the hallmark of chronic inflammation in IBD, but the exact role of neutrophils toward the host remained controversial [8–10]. Recently, clinical studies have identified elevated levels and densities of neutrophil extracellular traps (NETs) in IBD patients and correlated them with the disease severity [11–13]. NETs are DNA-based extracellular structures released by activated neutrophils during inflammation to capture pathogens and further activate the immune system. Typically, such NETs released during inflammation are destroyed by homeostatic activities of endogenous deoxyribonucleases (DNases) to prevent over-accumulation of NETs and inflammatory signals mediated by NETs [14]. Interestingly, the activities and levels of DNases were significantly impaired linked to the inability to degrade NETs in IBD patients [15, 16]. Although the exact mechanisms regarding the pathologic roles of NETs in IBD are not fully defined, DNase-I treatment in experimental settings has already shown promising outcomes against clinical manifestations of or disease associated with persistent NETs including SARS-CoV-2 and sepsis [17, 18]. Hence, recent findings regarding impaired neutrophil homeostasis in IBD patients [15, 16] greatly emphasize the potency of DNase-I as a therapeutic use for targeting NETs. Unfortunately, it is highly challenging to administrate free DNase-I to the colon owing to the low stability and short half-life of DNase-I in vivo [19]. Therefore, its clinical application requires promising approaches that could maximize its stability and activity even after administration.

**Scheme 1:**
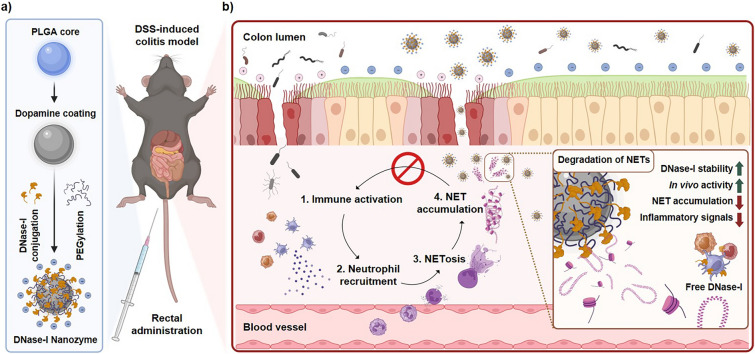
Schematic illustration of the fabrication of DNase-NZ and their application in the treatment of colitis. **a** DNase-NZ was fabricated using the synthetic polymer PLGA as the core. Polydopamine was used as a bio-adhesive coating for the conjugation of DNase-I. Nanozymes were PEGylated to improve their stability in vivo. **b** The negative surface charge of DNase-NZ allows passive recruitment to the damaged colon epithelium. Infiltrated particles attenuate NET-associated inflammations by inhibiting NET accumulation and interrupting the immune cascade. The inset demonstrates the advantage of DNase-NZ. With improved stability and enzyme activity, DNase-NZ can successfully degrade NET structures at inflamed sites while free DNase-I is rapidly cleared from the host environment. Scheme created using BioRender.com. PLGA, poly(lactic-co-glycolic acid); DSS, dextran sodium sulfate; NET, neutrophil extracellular trap

Herein, this study reports the use of nanoparticulate DNase-I, or DNase-I nanozyme (DNase-NZ), as an effective and stable platform for the treatment of IBD (Scheme 1). The nanozymes were fabricated upon a poly lactic-co-glycolic acid (PLGA) core, which was coated with dopamine to form a bio-adhesive layer. DNase-I was immobilized on the surface of PEGylated polymeric nanoparticles (NPs) to preserve maximal enzymatic activity while increasing in vivo stability (Scheme 1a). When DNase-NZ was treated to dextran sulfate sodium (DSS)-induced animal model of IBD, various pathophysiological characteristics of IBD were improved by attenuating neutrophil infiltration and NETosis in the colon compared to those treated with free DNase-I or mesalamine (Scheme 1b). This study suggests that DNase-NZ is an innovative and promising nanoplatform for targeting NETs, consequently leading to prevention of inflammation in the colon and a possible therapy for IBD treatment.

## Materials and methods

### Materials

Dextran sulfate sodium (MW ca 40,000 Da, cat no. J63606) was purchased from Alfa Aesar. RIPA Lysis Buffer (cat no. 89900) and Halt™ Protease Inhibitor Cocktail (cat no. 87786) were purchased from Thermo Scientific™. Dopamine hydrochloride (cat no. H8502) and Phorbol 12-myristate 13-acetate (PMA, cat no. 8139) were purchased from Sigma Aldrich. DNase-I (cat no. 10104159001), Collagenase D (cat no. COLLD-RO), and 1,4-dithiothreitol (DTT, cat no. DTT-RO) were purchased from Roche. Mesalamine (cat no. PHR1060) was purchased from Supleco. TRIzol™ Reagent (cat no. 15596026), RNAlater™ Stabilization Solution (cat no. AM7020), DNase I Buffer (cat no. AM8170G), Ambion™ DNase I (cat no. AM2222), and UltraPure™ Salmon Sperm DNA Solution (cat no. 15632011) were purchased from Invitrogen™. Percoll™ (Density 1.130 g/mL, cat no. 17089101) was purchased from Cytiva. Poly(lactic-co-glycolic acid) (50/50 ratio, inherent viscosity 0.55–0.75, acid terminated, cat no. B6013-2P) was purchased from LACTEL® Absorbable Polymers. 4arm polyethylene glycol (HCL salt, MW 5000, cat no. 4ARM-NH_2_ 5000) was purchased from JenKem Technology.

### Animals

All animal experiments were performed in accordance with the Guidelines for the Care and Use of Laboratory Animals established by the IACUC of Sungkyunkwan University (SKKU) Laboratory Animal Research Center (Suwon, Korea; Grant No: SKKUIACUC2023-02-16-1). C57BL/6 mice (female, 6–7 weeks old) were purchased from Orient Bio (Seongnam, Korea). All animals were housed under a 12:12 light–dark cycle and provided normal rodent pellets and drinking water ad libitum unless stated otherwise. All animals were acclimatized for 7 days before any experiments.

### Dextran sulfate sodium-induced colitis model

Prior to the induction of colitis, all animals were re-grouped according to their weight to ensure minimal weight variance across cages. Acute colitis was induced following a conventional method using dextran sulfate sodium (DSS) [20]. Mice were provided 2.5% DSS dissolved in drinking water ad libitum for 5 days. From day 5 onward, the water supply was changed to normal drinking water until the denoted day of killing.

### Colon lysate preparation

Colon samples were snap-frozen with liquid nitrogen and stored at − 80 ℃ before analysis. The distal portion of the colon was minced in RIPA buffer with protease inhibitors, then homogenized at 6000 rpm for 1 min using the Precellys® Evolution tissue homogenizer (Bertin Technologies, France). After centrifugation (10,000 × *g*, 20 min, 4 ℃), protein concentrations in the supernatant were quantified and normalized using the Pierce™ BCA protein assay kit (Thermo Scientific, USA). Lysate samples were immediately stored at − 80 ℃ until further analysis.

### Multiplex biomarker analysis

Colon lysates from mice killed on day 10 were used for biomarker analyses. The multiplexing analysis was performed using the Luminex™ 200 system (Luminex, Austin, TX, USA) by Eve Technologies Corp. (Calgary, Alberta).

### Plasma collection

Blood was collected from the submandibular veins of mice using a 4 mm Goldenrod animal lancet (MEDIpoint, USA) according to a published protocol [21]. Precise volumes of collected blood (25–50 µL) were recorded and immediately mixed with 25 µL of buffered sodium citrate (0.109 M) to prevent coagulation. Blood samples mixed with sodium citrate were centrifuged (1500 × *g* for 15 min at 4 ℃) to isolate the plasma. Isolated plasma was immediately stored at − 80 ℃ until further analysis. Blood samples were collected on days 0, 3, 6, 9, and 12 from alternating cheeks of mice.

### Enzyme-linked immunosorbent (ELISA) assay

DNase-I levels were analyzed from blood plasma using the Mouse Deoxyribonuclease I ELISA kit (MyBiosource, USA) following the manufacturer’s protocols. IL-1β and IL-6 levels were analyzed from colon lysates using the Mouse IL-1β DuoSet ELISA kit and Mouse IL-6 DuoSet ELISA kit (R&D Systems, USA) following the manufacturer’s protocols, respectively. All assays were performed in at least duplicates.

### Preparation of DNase-NZ

DNase-NZ was fabricated according to previously reported protocols [18, 22, 23] with slight modifications. The poly lactic-co-glycolic acid (PLGA) core was fabricated using the single emulsion method. PLGA (200 mg) was dissolved in 5 mL dichloromethane and mixed with 10 mL of 1% (w/v) polyvinyl alcohol. The mixture was sonicated (40% amplitude, 10 min, 1 s on–1 s off) using the Q700 sonicator (Qsonica, USA) and evaporated overnight under mild stirring. Bare PLGA particles were collected by centrifugation (17,000 rpm, 10 min, 4 ℃) and then washed three times with deionized water (PLGA NPs). The dopamine layer was coated by resuspending PLGA NPs in 10 mL Tris–HCl buffer (10 mM, pH 8.5) with dopamine hydrochloride (100 mg) under rapid stirring at 4 ℃ for 3 h. The dopamine-coated particles were collected by centrifugation (17,000 rpm, 10 min, 4 ℃) and then washed three times with deionized water (Dopa@PLGA NPs). PEGylated DNase-I nanozymes were fabricated by resuspending the dopamine-coated particles in 5 mL Tris–HCl buffer (10 mM, pH 8.5) with 50 mg DNase-I (Roche) and 50 mg poly(ethylene glycol). The mixture was vigorously stirred overnight at 4 ℃, centrifuged (17,000 rpm, 10 min, 4 ℃), and then washed thrice with deionized water (DNase-NZ). PEGylated NPs without surface DNase-I were prepared using the identical method used to fabricate DNase-NZ without adding DNase-I (PEG@D-PLGA NPs).

### Characterization of DNase-NZ

The size, polydispersity index, and zeta potential of particles at each fabrication step were measured using the Zetasizer Pro (Malvern Instruments, USA). The morphology of each particle was analyzed and imaged using the JSM-IT800 scanning electron microscope (JEOL, Japan) at a magnification of 100,000×.

### Assessment of enzymatic activity and stability of DNase-NZ

In this study, we defined one enzymatic unit as the amount of enzyme required to completely degrade 1 µg of DNA in 10 min at 37 ℃. According to this definition, one unit of DNase-NZ or free DNase-I was experimentally determined by performing a DNA degradation assay across serially diluted samples. In inI buffer (2 µL), salmon sperm DNA (1 µg), and test material for 10 min at 37 ℃. 1 U of DNase-I (Invitrogen) was added instead of test materials when preparing a positive control. The incubated mixtures were electrophoresed on a 1% agarose gel stained with ethidium-bromide (0.5 µg/mL). The presence or absence of DNA bands was visualized using the ChemiDoc™ XRS + system (Bio-Rad Laboratories, USA), equipped with standard excitation and emission filters.

To assess the stability of DNase-NZ, particles were dispersed at a concentration of 100 U/mL in a simulated biological fluid composed of phosphate-buffered saline (PBS) (1X, pH 7.4) and 10% fetal bovine serum (FBS) and incubated in a shaking incubator (37 ℃, 100 rpm) for prolonged durations (0, 1, 3, 6, 12, 24, 48 h). Sample volume equivalent to one unit of DNase-NZ (10 µL) was isolated at each time point, centrifuged, washed, and then assessed using the DNA degradation assay to determine whether the DNase-NZ maintained its enzymatic activity post-incubation.

### Cytotoxicity assay

L929 cells purchased from the Korean Cell Line Bank (KCLB) were cultured in complete DMEM in a humidified incubator (37 ℃, 5% CO_2_). Before the assay, cells were seeded to 96-well plates (5 × 10^4^ cells per well) and incubated for 24 h. After removal of culture media, fresh media (100 µL) containing different concentrations of DNase-NZ (0, 0.5, 1, 2, 5, 10, 50, 100, 500, 1000 µg/mL) was added to each well accordingly. After 24 h incubation, the cytotoxicity of DNase-NZ was evaluated using the D-Plus™ CCK cell viability assay kit (Dongin LS, South Korea), following the manufacturer’s protocols.

### Degradation of neutrophil extracellular traps in vitro

NETs were induced from bone-marrow derived neutrophils and quantified according to a previous study [24] with slight modifications. Briefly, bone marrow cells were harvested from the femur and tibia of mice, and then purified using a three-phase Percoll gradient (55%, 60%, 80%). Neutrophils at the 60%/80% Percoll interface were isolated, washed, and counted using the LUNA-II cell counter (Logos Biosystems, South Korea). Immediately after seeding neutrophils in a black 96-well plate (1 × 10^5^ cells per well), NETosis was induced by adding 30 µL of PMA (2 µM stock) per well. After 3 h of incubation, free DNase-I (Invitrogen, 5 U) or DNase-NZ (5 U) was added to degrade NETs. After a further 45 min, 30 µL of SYTOX Green (50 µM stock) was added to each well and incubated for 15 min to stain extracellular DNA. Fluorescence (Ex. 488 nm / Em. 523 nm) was measured and compared using the SpectraMax iD3 microplate reader (Molecular Devices, USA). The percentage of DNA released by PMA was calculated using the following formula:

$$\mathrm{Percentage \, DNA \, induced \, by \, PMA}=\frac{\mathrm{ Intensity }\left({\text{BMDN}}+{\text{PMA}}\right)-\mathrm{ Intensity }\left({\text{BMDN}}+{\text{PMA}}+{\text{DNase-I or DNase-NZ}}\right)}{\mathrm{ Intensity }\left({\text{BMDN}}+\mathrm{Triton X-100}\right)}$$

### Treatment of DSS-induced colitis model

Five experimental groups (5 mice per group) were compared in this study. Four groups were induced with acute colitis by providing 2.5% DSS for 5 days and normal water thereafter, while the control group was only provided with normal water. Colitis mice were daily administered with either PBS (200 µL), mesalamine (100 mg/kg), free DNase-I (Roche, 500 U in 200 µL PBS) or DNase-NZ (500 U in 200 µL PBS) intra-rectally once a day for 7 days (D0 to D6). Control mice were also daily administered with PBS for 7 days. Changes in body weight were recorded daily. The disease activity index was also assessed referring to parameters reported in a previous work [25] (Table 1). On day 10, all mice were killed for further analysis. Colons were excised after killing to measure the colon length and various portions of the colon, excluding the cecum were used for subsequent analyses (Most proximal – myeloperoxidase (MPO) activity; Proximal – flow cytometry; Middle – ELISA; Distal – RNA isolation; Most distal – histology). Spleen weight was measured immediately after killing to calculate the spleen index (Spleen weight in mg / Body weight in g).

**Table 1:** Scoring parameters used to assess the disease activity index

Score	Stool consistency	Bleeding	Weight loss
0	Formed	Normal color	No weight loss
1	Mild soft	Brown color stool	1–5% weight loss
2	Very soft	Reddish color stool	6–10% weight loss
3	Watery	Bloody stool	11–15% weight loss
4	–	–	≥ 16% weight loss

### MPO activity assay

Colon samples were snap-frozen with liquid nitrogen and stored at − 80 ℃ prior to analysis. MPO activity was analyzed using a published protocol [26] with slight modifications. In brief, HTAB buffer (13.7 mM; 50 mM potassium phosphate buffer) was added to each colon sample at a ratio of 12.5 mg/mL and homogenized (6000 rpm, 1 min) using the Precellys® Evolution tissue homogenizer (Bertin Technologies, France). The supernatant was collected and stored in ice after centrifugation (18,000 × *g*, 15 min, 4 ℃). In a 96-well plate, 7 µL of tissue homogenates were mixed with 50 µL of 0.05% H_2_O_2_ solution and 200 µL of o-dianisidine solution (526 µM; 5 mM potassium phosphate buffer). Two absorbances (abs. 460 nm) at 1 min intervals were immediately measured using the SpectraMax iD3 microplate reader (Molecular Devices, USA). The MPO activity of each tissue sample was calculated following a published protocol [26].

### Histological analysis

Colon samples were fixed in 4% paraformaldehyde for 24 h at 4 ℃ and embedded in paraffin blocks before histological analyses. Prepared paraffin blocks were sectioned (5 µm thickness) and stained with either Hematoxylin and Eosin (H&E) or Alcian blue/Periodic Acid-Shiff (AB/PAS) for analyses. Histologic scores were also carefully assessed in a blinded fashion referring to parameters reported in a previous work [27]. Briefly, histological scores were assigned according to the following: 0, normal; 1, hyperproliferation, irregular crypts, and goblet cell loss; 2, mild to moderate crypt loss (10–50%); 3, severe crypt loss (50–90%); 4, complete crypt loss, surface epithelium intact; 5, small- to medium-sized ulcers (< 10 crypt widths); 6, large ulcers (≥ 10 crypt widths). Inflammatory cell infiltration was scored separately for the mucosa (0, normal; 1, mild; 2, modest; 3, severe), submucosa (0, normal; 1, mild to modest; 2, severe), and muscle/serosa (0, normal; 1, moderate to severe) (Table 2). All scores were summed together resulting in a maximum total score of 15.

**Table 2:** Scoring parameters used to assess histological score

Score	Colonic damage	Cell infiltration (Mucosa) / (submucosa) / (serosa)
0	Normal	Normal
1	Hyperproliferation, irregular crypts, and goblet cell loss	Mild
2	Mild to moderate crypt loss (10–50%)	Modest
3	Severe crypt loss (50–90%)	Severe
4	Complete crypt loss, surface epithelium intact	–
5	Small- to medium-sized ulcers (< 10 crypt widths)﻿	–
6	Large ulcers (≥ 10 crypt widths)	–

### Isolation of lamina propria mononuclear cells

Colon samples were briefly stored in ice-cold PBS and immediately used to isolate lamina propria mononuclear cells (LPMCs). To isolate cells from the lamina propria, the colon was cut longitudinally and thoroughly washed of any fecal contents with ice-cold HBSS. Tissue samples were cut into 1 cm pieces and incubated with pre-digestion solution (1X HBSS, 2 mM EDTA, 1 M DTT, 10 mM HEPES) in a shaking incubator (37 ℃, 100 rpm) for 20 min to separate the epithelial layer. The remaining tissue was thoroughly washed with ice-cold PBS. Afterward, the colon was mechanically minced with scissors and digested in a solution of 0.5 mg/mL Collagenase D and 20 µg/mL DNase-I (Roche) dissolved in complete RPMI 1640 medium. Samples were incubated in a shaking incubator (37 ℃, 100 rpm) for 90 min and filtered through a 40 µM cell strainer to remove undigested tissue. Isolated lamina propria cells were centrifuged (400 × *g*, 10 min), and washed with PBS twice before further analysis.

### Flow cytometry analysis

Each flow cytometry analysis used 2 × 10^6^ isolated lamina propria cells. All samples were first stained with Fc blocking antibody for 10 min at 4 ℃ in the dark to inhibit non-specific Fc receptor bindings. Cells were gently washed and further stained using diluted antibodies for 20 min at 4 ℃ in the dark. Cells were gently washed, resuspended in 1 mL PBS, and used for analysis on the CytoFLEX flow cytometer (Beckman Coulter, USA). All antibodies were diluted in PBS containing 2% FBS. The following antibodies were used in this study: CD16/CD32 Mouse Fc Block™ (dil. 1:500) (BD Pharmingen™, USA), Zombie Violet™ Viability Dye (dil. 1:500), CD45 (QA17A26; FITC, dil. 1:200), CD11c (N418; PerCP-Cy5.5, dil. 1:400), Ly6G (1A8; BV650, dil. 1:400) (BioLegend, USA). Data analysis was performed using the CytExpert software provided by Beckman Coulter.

### RNA isolation and quantitative real-time PCR

Colon samples were stored in RNAlater solution and stored at -20 ℃ prior to analysis. Total RNA from colon tissues was isolated using the TRIzol® reagent. cDNA was synthesized using the High Capacity cDNA Reverse Transcription Kit (Applied Biosystems, USA) according to the manufacturer’s protocols. Real-time qPCR analysis was performed on the StepOnePlus Real-Time PCR System (Applied Biosystems, USA) using the PowerUP™ SYBR™ Green Master Mix (Applied Biosystems, USA). The following primer sequences were used in this study: *Gapdh* forward 5’-GAACGGATTTGGCCGTATTG-3’, reverse 5’-GTTGAATTTGCCGTGAGTGG-3’; *Elane* forward 5’-CTACTGGCATTGTTCCTGGG-3’, reverse 5’-CCACAGAAATGACCTCCACG-3’; *Padi4* forward 5’-GGATGCAGGACGAAATGGAG-3’, reverse 5’-GGACCCATAACTCGCTTGAC-3’; *Cxcl5* forward 5’-ACGGTGGAAGTCATAGC-3’, reverse 5’-TGAACACTGGCCCTTCTTTC-3’. Gene expression was normalized to the housekeeping gene *Gapdh*. Relative expression was calculated using the ΔCt method as the value of 2^–ΔCt^.

### Statistical analysis

Data were presented as mean ± standard error (SEM) with a minimum of three samples unless stated otherwise. All analyses were performed in at least duplicates. The precise number of replicates or samples (*n*) for each data was mentioned in the figure descriptions. Statistical significance was assessed using the unpaired Student’s *t*-test in GraphPad Prism software. P-values of *p < 0.0332, **p < 0.0021, ***p < 0.0002, and ****p < 0.0001 were considered statistically significant. The precise statistical method and P-values are mentioned in the figure descriptions.

## Results and discussion

### Biomarker analysis of colitis mice

**Fig. 1:**
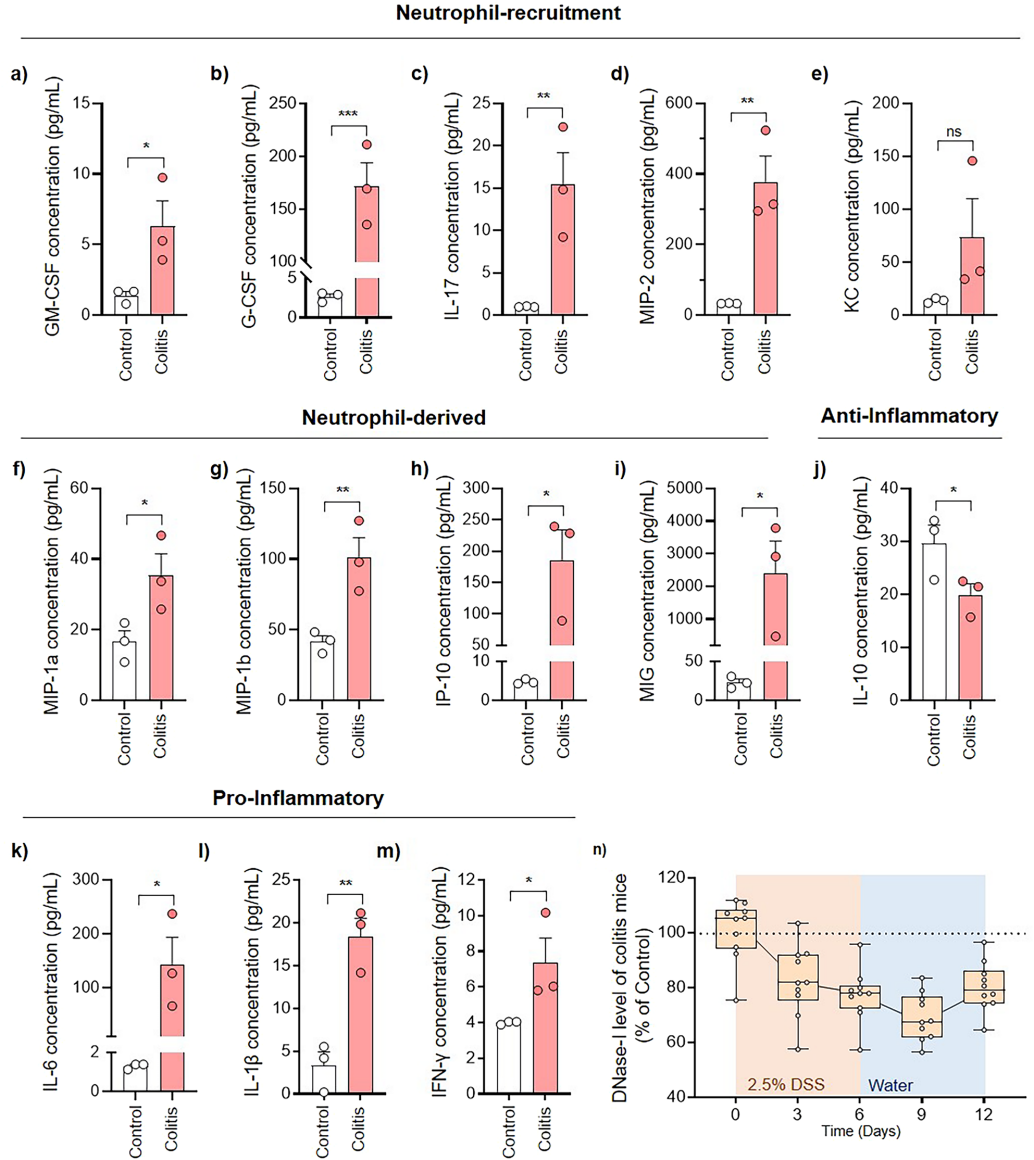
Changes in various neutrophil-related biomarkers during acute colitis in C57BL/6 mice. **a-m** Biomarker levels were analyzed from the colon of normal and colitis mice (n = 3 per group). **a** GM-CSF, **b** G-CSF, **c** IL-17, **d** MIP-2, **e** KC, **f** MIP-1, **g** MIP-1, **h** IP-10, **i** MIG, **j** IL-10, **k** IL-6, **l** IL-1β, and **m** IFN-γ levels were simultaneously analyzed using the Luminex® assay. Biomarkers were categorized as either **a**–**e** those involved in neutrophil recruitment, **f**–**i** those released from activated neutrophils, **j** anti-inflammatory, or **k**–**m** pro-inflammatory. **n** Changes in plasma DNase-I levels during the development of acute colitis (n = 10). DNase-I levels in normal mice were averaged and used as the baseline (100%) to calculate relative changes in colitis mice. All analyses were performed in duplicates. Data are presented as mean ± SEM **a-m** or min./median/max. **n** Statistical significance was assessed using a one-tailed Student’s t-test. *p < 0.0332, **p < 0.0021, ***p < 0.0002

Many murine colitis models are currently available for the study of IBD; however, no model can fully mimic the precise pathogenesis of the actual disease [28]. Therefore, before directly assessing the therapeutic effects of DNase-I, a preliminary study was performed to briefly understand the neutrophil-related pathogenesis in a conventional IBD model. To elucidate the role of neutrophils and NETs in the development of a DSS-induced colitis model, a multiplex analysis was conducted upon a range of biomarkers (Fig. 1). For this study, two major groups of biomarkers were selected: those involved in NETosis or neutrophil recruitment (i.e., GM-CSF, G-CSF, IL-17, MIP-2, KC), and those secreted by activated neutrophils (i.e., MIP-1α, MIP-1β, IP-10 and MIG). Colitis was induced in female C57BL/6 mice by providing 2.5% DSS dissolved in water for five days. The colons of mice were excised on day 10 for the following analyses. In DSS-induced colitis mice, colon levels of GM-CSF, G-CSF, IL-17, and MIP-2, KC were highly elevated compared to control mice (Fig. 1a–e). GM-CSF, G-CSF and IL-17 are factors that cohesively recruit [29, 30] and prime neutrophils for NET release [31]. On the other hand, MIP-2 [32] and KC [33, 34] are chemokines released from intestinal epithelial cells during microbial infection to further recruit and activate neutrophils. Furthermore, colons of colitis mice also exhibited significantly elevated levels of MIP-1α, MIP-1β, IP-10, and MIG (Fig. 1f–i). Given that these neutrophil-derived chemokines also play roles as chemotactic and activating factors against other immune cells, such as macrophages, natural killer cells, and dendritic cells [35], our findings suggest that activated neutrophils indeed play an integral role in immune modulation that affects the development of DSS-induced colitis.

In line with such findings, the level of anti-inflammatory cytokine IL-10 was highly suppressed in colitis mice (Fig. 1j), whereas pro-inflammatory cytokine levels (IL-6, IL-1β, IFN- γ) were significantly elevated (Fig. 1k–m). This indicated that the intestinal environment in DSS-induced colitis mice was shifted towards a pro-inflammatory state with suppressed anti-inflammatory cytokines. Plasma DNase-I levels were observed throughout the course of colitis development to investigate whether NET degradation is impaired in colitis mice. Consistent with the development of colitis, plasma DNase-I levels were increasingly downregulated in colitis mice compared to those of control mice (Fig. 1n). Interestingly, DNase-I levels in colitis mice slightly increased on day 12 (80.17% of normal) compared to day 9 (69.15% of normal). Considering that the body weight of colitis mice also started to increase after day 10 (Additional file 1: Fig. S1), the increase in DNase-I level may be explained as the result of natural recovery following the termination of DSS administration. The observations herein do not provide a comprehensive interpretation of the NET-induced pathogenesis of IBD. Nevertheless, our findings demonstrated elevated levels of neutrophil/NET-related biomarkers and reduced DNase-I levels during colitis development, which collectively provides a promising rationale for the use of DNase-I as a potential therapeutic enzyme against IBD.

### Fabrication and characterization of DNase-NZ

**Fig. 2:**
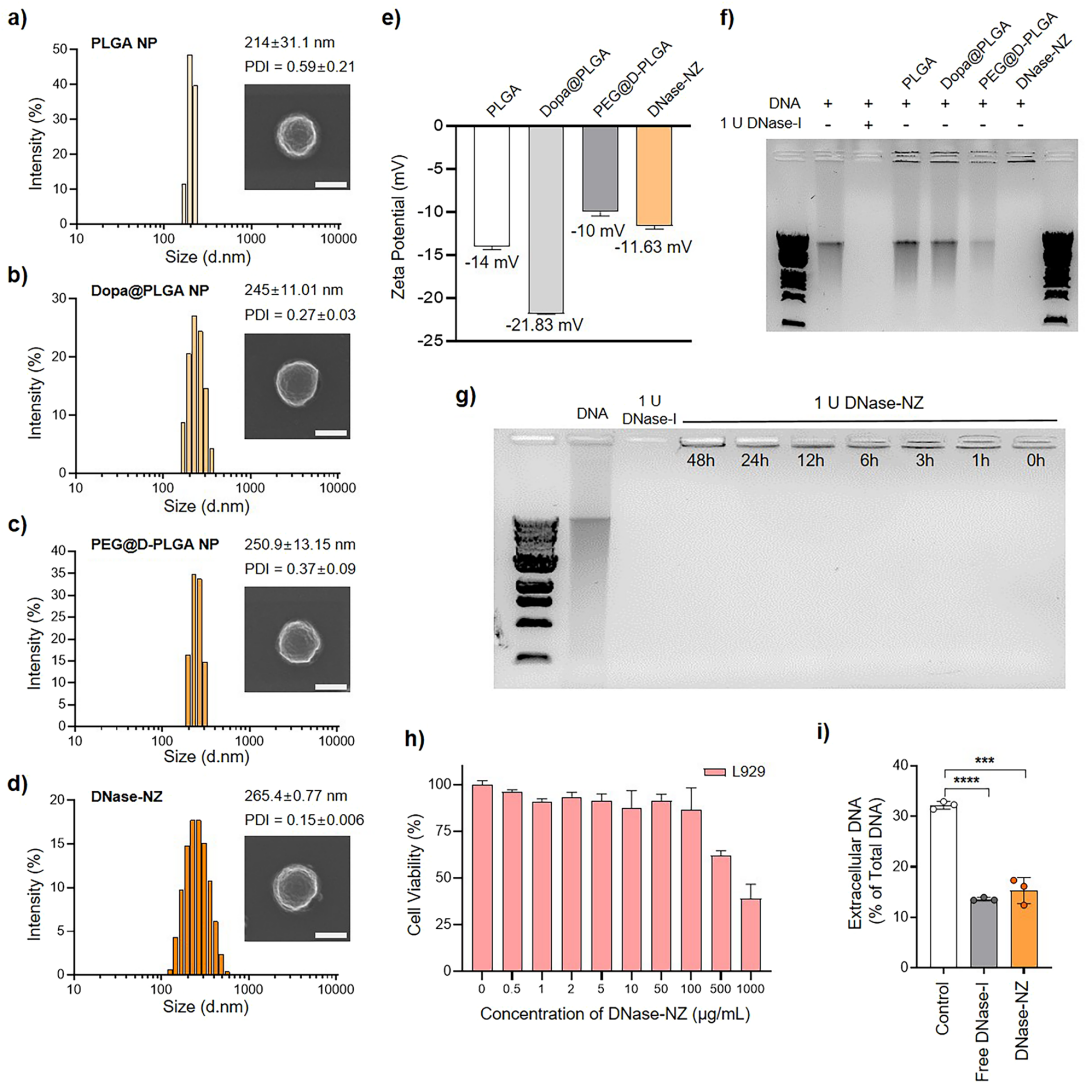
Characterization of DNase-NZ. **a**–**d** Size distribution, average diameter, and polydispersity index of **a** PLGA NPs, **b** Dopa@PLGA NPs, **c** PEG@D-PLGA NPs, and **d** DNase-NZ. **a**–**d** Insets represent a scanning electron micrograph of each particle imaged at 10,000× magnification. Scale bar: 200 nm. **e** Surface charges of PLGA NPs, Dopa@PLGA NPs, PEG@D-PLGA NPs, and DNase-NZ. **f** The enzymatic activity of DNase-NZ was confirmed using gel electrophoresis. **g** The enzymatic activities of DNase-NZ were successfully preserved after prolonged incubations (1, 3, 6, 12, 24, and 48 h) at 37 ℃. **h** In vitro cytotoxicity of DNase-NZ on L929 cells at various concentrations (0.5, 1, 2, 5, 10, 100, 500, and 1000 μg/mL) after 24 h incubation. **i** NET-degrading ability of DNase-NZ was confirmed after incubation with NETosis-induced bone marrow derived neutrophils (BMDNs). Data represented as mean ± SD. **e**, **h**, **i** Statistical significance was assessed using a two-tailed Student’s t-test. ***p < 0.0002, ****p < 0.0001

DNase-I is an endogenous enzyme that effectively degrades NETs released from neutrophils during inflammation. However, freely administered DNase-I is highly unstable and susceptible to rapid clearance in vivo, making traditional DNase-I treatments impractical against IBD. Accordingly, referring to our previous publication [18], DNase-I was immobilized on the surface of a polymeric core to fabricate polymeric nanozymes with enhanced enzymatic stability and in vivo activity (Scheme 1a). The PLGA core was initially fabricated using the single emulsion method. The size of PLGA nanoparticles (PLGA NPs) measured 214 ± 31.1 nm in diameter, and exhibited spherical morphologies confirmed using scanning electron microscopy (Fig. 2a). PLGA NPs were then readily coated with dopamine by suspending PLGA NPs in Tris–HCl buffer with dopamine hydrochloride under rapid stirring for 3 h to form an intermediate coupling layer [36]. Owing to its rich amine and catechol functional groups, the dopamine coating allows covalent conjugation of various thiols and amines via Michael addition or Schiff base reactions [37]. Dopamine-coated NPs (Dopa@PLGA NPs) were further functionalized with only PEG (PEG@D-PLGA NP) or both PEG and DNase-I in a one-pot process to fabricate DNase-NZ. Dopa@PLGA NPs were again simultaneously suspended in Tris–HCl buffer with PEG and free DNase-I under rapid stirring overnight to ensure sufficient reaction between the surface dopamine and PEG/DNase-I. The diameters of Dopa@PLGA NPs, PEG@D-PLGA NPs, and DNase NZs were each 245 ± 11.01 nm, 250.9 ± 13.15 nm, and 265.4 ± 0.77 nm, respectively (Fig. 2b–d). The sizes of the particles fabricated in this study were slightly larger than those reported previously [18] but still successfully demonstrated a sequential increase after each functionalization. Furthermore, scanning electron micrographs confirmed that the spherical morphologies were successfully maintained throughout each fabrication process (Fig. 2b–d). The monodispersity of DNase-NZ was confirmed via its low polydispersity index (0.15 ± 0.006). While all nanofabrications exhibited negative surface charges, the surface charge of DNase-NZ (− 11.63 ± 0.37 mV) was lower than those of PEG@D-PLGA NPs (− 10 ± 0.45 mV), likely owing to the presence of DNase-I (Fig. 2e). Multiple studies have reported the use of PEG and dopamine to enhance mucopenetration while minimizing the mucoadhesion of nano-sized materials [38–40]. Interestingly, unlike the normal intestine, damaged regions of the intestinal epithelium are known to become positively charged [41]. Therefore, the PEG and dopamine coatings not only allow DNase-NZ efficient mucopenetration, but the negative surface charge may also allow enhanced interaction of DNase-NZ to damaged colon areas due to electrostatic attraction.

Next, the enzymatic activity of DNase-NZ was evaluated using a DNA degradation assay (Fig. 2f). Unlike those of small molecular drugs, the therapeutic effects of immobilized enzymes may vary depending on its orientation during immobilization [42, 43]. Therefore, many studies involving immobilized DNase-I have conventionally used enzymatic units as the quantitative measurements of functional enzymes [18, 22, 23]. For standardization purposes, we also used enzymatic units as the practical measurement of DNase-I, and in this study, one unit (U) of DNase-I activity was defined as the amount of nanozyme required to successfully degrade 1 µg of DNA in 10 min when incubated at 37 ℃. The unit-equivalent amount of DNase-NZ was determined by performing a DNA degradation assay across serially diluted DNase-NZ samples (0.5–25 µg) (Additional file 1: Fig. S2). 1 U was determined as 2.5 µg of lyophilized DNase-NZ, and this measurement was applied throughout all following unit calculations. Figure 2f represents the DNA degradation capacity of different particle formulations when added at amounts equivalent to one unit of DNase-NZ (2.5 µg). Of all nanofabrications, only DNase-NZ could successfully degrade DNA, owing to its unique presence of surface DNase-I. Like other protein drugs, exogenous DNase-I is highly susceptible to degradation and/or reduction in activity by proteases, clearance mechanisms, and conformational changes when administrated freely [44, 45]. However, considering that immobilization and PEGylation provide increased operational stability, thermal stability, and resistance to systemic clearance [46], we predicted that DNase-NZ could stably preserve its enzymatic activity. To test the enzymatic stability of DNase-NZ in vitro, the nanozymes were incubated in a simulated biological fluid at 37 ℃ for prolonged durations (1, 3, 6, 12, 24, and 48 h). Using the aforementioned DNA degradation assay, we confirmed that DNase-NZ successfully preserved their enzymatic activities even after 48 h (Fig. 2g). Previous studies have demonstrated that nanoparticles about 200 nm in diameter fabricated using 50:50 ratio PLGA significantly degrade three days after administration in vivo [47, 48]. Considering the biodegradability of the PLGA polymer, we concluded that 48 h of sustained enzymatic activity were sufficient for further in vivo applications. Meanwhile, the maximum degradation time proposed by the manufacturers of the PLGA polymer used in this study is less than 3 months.

### Cytotoxicity of DNase-NZ

The cytotoxicity of DNase-NZ was determined against mouse fibroblast cells (L929 cells) using a commercial cell viability assay kit (Fig. 2h). Cells were directly treated with various concentrations of DNase-NZ (0.5–1000 µg/mL) for 24 h. L929 cells did not exhibit any signs of toxicity and maintained high viability (> 90%) against DNase-NZ at concentrations up to 100 µg/mL (12.5 U/cm^2^). At higher concentrations, the viability of cells started to decrease rapidly, making 100 µg/mL the maximal biocompatible dose in vitro. Safe doses of DNase-NZ for in vivo application were separately calculated regarding both the presented cytotoxicity of the nanozyme and the surface area of the murine colon [49].

### Degradation of NETs produced by neutrophils in vitro

Apart from the enzymatic activity of DNase-NZ confirmed against free DNA (Fig. 2f), the activity of DNase-NZ against NETs was further analyzed in vitro (Fig. 2i). For this assay, neutrophils were isolated from the bone marrow of C57BL/6 mice (bone marrow-derived neutrophils [BMDNs]) as reported in a previous study [24]. NETosis was induced in BMDNs using 200 nM PMA as the stimulant. To quantify NETs, the extracellular DNA released during NETosis was stained with SYTOX green, a membrane-impermeable fluorescent dye specific to extracellular nucleic acids. The total DNA content measured from fully dead/lysed cells was used as a reference to calculate the relative amount of extracellular DNA released as the result of NETosis. In BMDNs treated with only PMA, the amount of extracellular DNA released was 32.23 ± 0.74% of total DNA. When 2.5 U DNase-NZ was added to NETosis-induced BMDNs, the percentage of extracellular DNA was significantly reduced to 15.33 ± 2.58%. As we expected, the reduction in extracellular DNA in vitro was similar to those observed using free DNase-I (13.62 ± 0.33%). Our data indicate that the enzymatic activities of DNase-NZ are highly effective towards NETs produced by activated neutrophils.

### Therapeutic efficacy of DNase-NZ against DSS-induced colitis

**Fig. 3:**
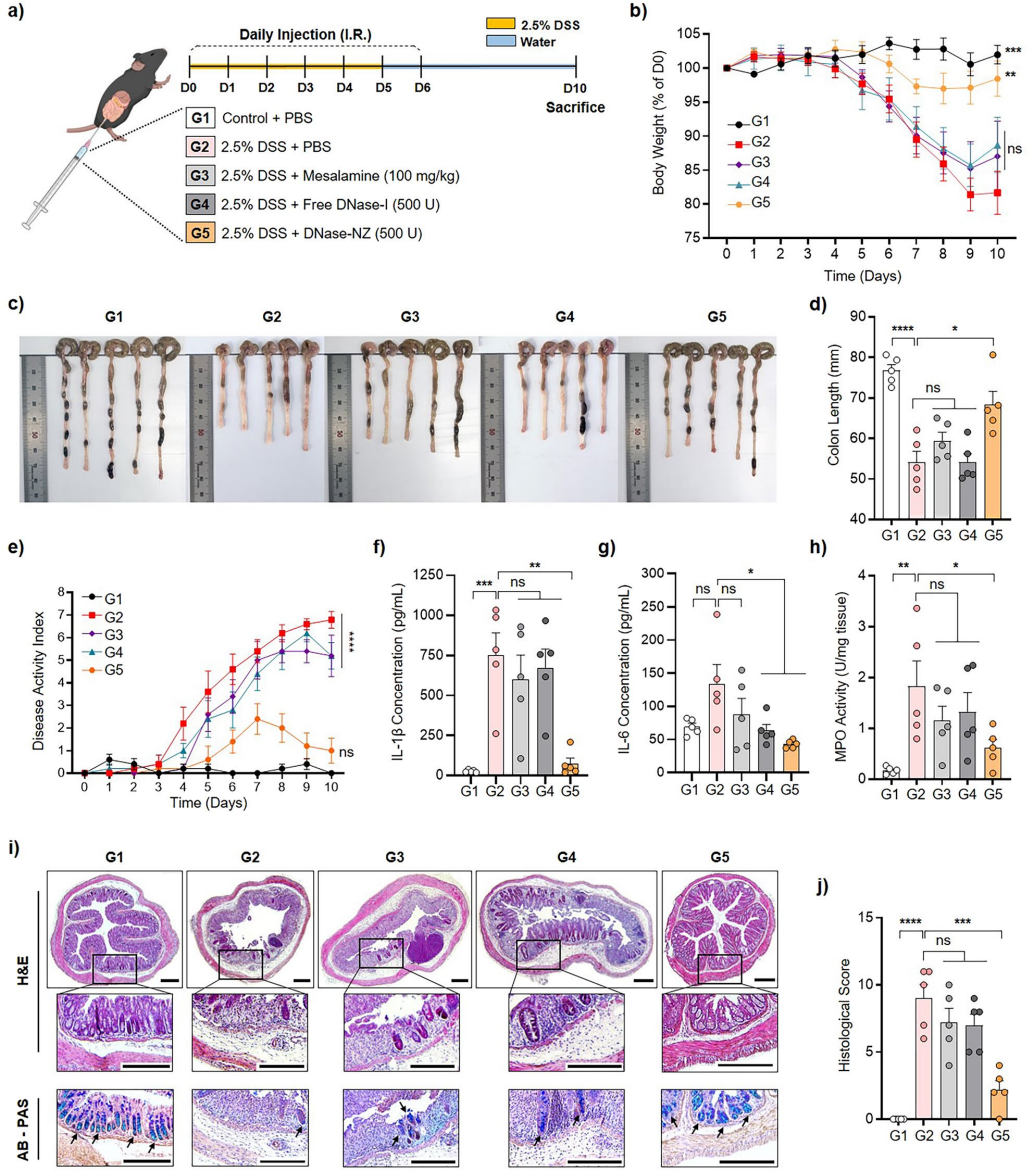
Therapeutic efficacy of DNase-NZ against DSS-induced colitis in mice. **a** Scheme illustrating the experimental groups and treatment schedule. C57BL/6 mice were provided with 2.5% DSS dissolved in drinking water for five days to induce acute colitis. PBS, mesalamine (100 mg/kg), free DNase-I (500 U), or DNase-NZ (500 U) was intra-rectally administrated daily for 7 days (Day 0–6). All mice were killed for analysis on day 10. **b** Changes in body weight recorded daily (n = 5). Statistical significance was assessed against G2. **c**, **d** Image and quantitative measurement of colon lengths on day 10 (n = 5). **e** Disease activity index (DAI) scored daily based on weight loss, stool consistency, and rectal bleeding (n = 5). Statistical significance assessed against G2 **f**, **g** Quantitative analysis of pro-inflammatory cytokine levels including **f** IL-1β and **g** IL-6 using commercial ELISA kits (n = 5). **h** Quantitative analysis of colon MPO activity on day 10 (n = 5). **i** Representative H&E and AB-PAS staining images of colon tissues on day 10. Arrows in AB-PAS stained images indicates presence of mucin. Scale bars: 250 µm. **j** Histological scores based on microscopic appearance of H&E stained images (n = 5). Data are presented as mean ± SEM. Statistical significance was assessed using two-tailed Student’s t-test. *p < 0.0332, **p < 0.0021, ***p < 0.0002

The therapeutic effects of DNase-NZ were assessed using a DSS-induced animal model of colitis. To induce acute colitis, female C57BL/6 mice were provided with 2.5% DSS dissolved in drinking water for 5 days, then normal water for 5 days (Fig. 3a). Before this study, pilot experiments were conducted to determine the therapeutic dose of DNase-NZ. The rectal route was used in this study to achieve colon-specific localization of DNase-NZ. Interestingly, when colitis mice were treated with DNase-NZ doses (50, 100, and 200 U) that were reported as effective against other diseases [18, 23], the treatments did not exhibit any notable therapeutic effects (Additional file 1: Fig. S3). This may be due to the difference in choice of administration route across studies. Unlike particulate DNase-I in other studies that were administered intraperitoneally [22, 23], DNase-NZ administered intra-rectally must penetrate both the mucus and epithelial barrier to reach its site of action [50, 51], which could have critically reduced the delivery efficiency of DNase-NZ. The mucus barrier is commonly destroyed during IBD [52] and PEGylation provides further mucopenetration properties to particles [53], making mucopenetration of PEGylated DNase-NZ highly feasible after intrarectal administration in colitis models. Even though we have not directly observed the internalization mechanism of DNase-NZ against the colon epithelia, various studies upon nanoparticles of similar characteristics suggest that DNase-NZ is likely internalized via caveolae-mediated endocytosis [54] owing to its favorable size (265.4 ± 0.77 nm) and anionic surface charge (− 11.63 ± 0.37 mV).

We accordingly chose 500 U as the experimental dose for the following experiments. For the first 7 days, colitis mice were intra-rectally administered with either mesalamine (100 mg/kg), free DNase-I (500 U), or DNase-NZ (500 U) every day. Control mice were only provided with drinking water for the entire duration, and untreated colitis mice were administered with PBS instead. Mesalamine was included in this study for comparison as a control medicine used in IBD treatment [49]. Throughout the study, changes in body weight were monitored daily and used as the primary indicator of colitis development (Fig. 3b). Body weights of mice treated with either PBS, mesalamine, or free DNase-I started to rapidly decrease after day 4, eventually reaching 80.65 ± 2.96%, 84.56 ± 4.14%, and 85.18 ± 3.45% of original body weight, respectively, at day 9 (Fig. 3b, Additional file 1: Fig. S4a). Even though mesalamine or free DNase-I treatment could slightly attenuate body weight loss, the differences were negligible compared to those treated with DNase-NZ. Notably, the maximum weight loss of mice treated with DNase-NZ was 4.73 ± 1.68% of the original weight, highlighting the enhanced efficacy of the nanozyme platform compared to free DNase-I. DNase-NZ treatment also successfully protected mice from colon shortening compared to other treatments (Fig. 3c, d). The final colon lengths of the DNase-NZ group (68.30 ± 3.31 mm) were slightly shorter than those of normal mice (76.74 ± 1.50 mm); however, the extent of colon shortening was significantly attenuated compared to both the PBS (54.11 ± 2.71 mm) and the free DNase-I group (54.01 ± 2.14 mm). Interestingly, the disease activity index (DAI) of mice treated with DNase-NZ initially increased, after which the scores decreased to levels comparable to those of control mice by day 10 (Fig. 3e). The DAI scores of mice treated with either PBS, mesalamine, or free DNase-I remained constantly elevated, indicating the presence of active colitis. Next, we examined the levels of pro-inflammatory cytokines in the colon tissues using the ELISA assay. DNase-NZ treatment significantly reduced the levels of both IL-1β (Fig. 3f) and IL-6 (Fig. 3g). In particular, IL-1β levels in mice treated with DNase-NZ were decreased up to tenfold compared to PBS-treated colitis mice.

MPO is an enzyme abundantly degranulated by activated neutrophils, making it highly specific indicators of neutrophil-mediated inflammation and oxidative stress in the colon [55]. Accordingly, colonic MPO activity was assessed using a colorimetric method [26] (Fig. 3h). As expected, the level of MPO activity in colitis mice was significantly higher than those of control mice. However, mice treated with DNase-NZ showed significantly decreased levels of MPO activity (0.61 ± 0.16 U/mg) compared to mice either treated with PBS (1.83 ± 0.50 U/mg), mesalamine (1.15 ± 0.29 U/mg), or free DNase-I (1.33 ± 0.38 U/mg), indicating that DNase-NZ successfully inhibited intestinal inflammations. We also performed histological analyses of either H&E or AB/PAS stained colon sections, additionally confirming the therapeutic effect of DNase-NZ against DSS-induced colitis (Fig. 3i). From colon H&E samples of colitis mice treated with PBS, severe ulceration, destruction of crypts, and substantial infiltration of immune cells were observed. In contrast, DNase-NZ treatment was able to aid the restoration of the colon epithelium, evidenced by intact morphological structures (e.g., epithelium, mucosa, crypt) and minimal infiltration of inflammatory cells resembling conditions similar to those of control mice. AB/PAS stained colon slides of the DNase-NZ group also showed a notable restoration of goblet cells comparable to those of the control group. These morphological observations were further quantified and compared using a histological scoring system (Fig. 3j, Table 2).

### Effects of DNase-NZ on neutrophil infiltration and NETosis in DSS-induced colitis

**Fig. 4:**
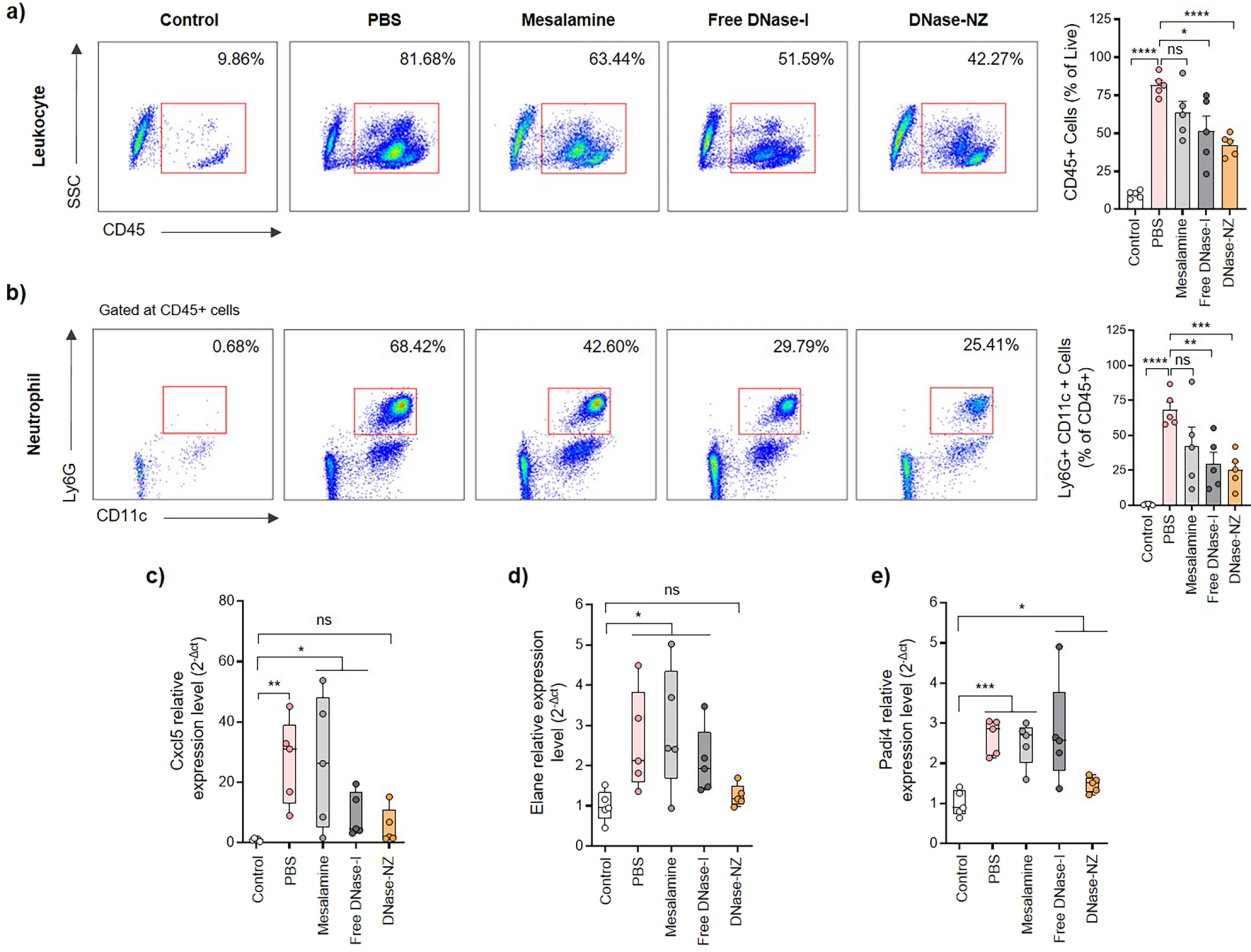
Changes in immune cell population and NETosis-related genes after treatment with DNase-NZ. **a** Representative flow cytometry plots (left) of leukocytes as identified by CD45^+^ cells in the lamina propria of mice (gated on live cells). Numbers within the plot indicate the average percentage of cells in the corresponding subset. The bar graph (right) represents the percentage of cells positive for CD45. (n = 5) **b** Representative FACS plots (left) of the neutrophils as identified by Ly6G and CD11c in the lamina propria of mice (gated on CD45 + leukocytes). Numbers within the plot indicate the average percentage of cells in the corresponding subset. The bar graph (right) represents the percentage of cells positive for Ly6G and CD11c. **c–e** Relative mRNA expression levels of genes, including **c** *Cxcl5*, **d** *Elane*, and **e** *Padi4*, were determined using quantitative PCR assay (n = 5). *Gapdh* was used as an internal reference and relative expression levels were calculated against control mice. All assays were performed in at least duplicates. Data are presented as mean ± SEM. Statistical significance was assessed using two-tailed Student’s t-test. *p < 0.0332, **p < 0.0021, ***p < 0.0002, ****p < 0.0001

To evaluate the effect of DNase-NZ administration on infiltrating neutrophils, immune cell populations were quantified using flow cytometry (Fig. 4a, b). Cells were first isolated from the lamina propria (LP) using an enzymatic method. Using CD45 as a pan-leukocyte marker, we identified the baseline population of leukocytes in the LP of control mice as 9.86 ± 1.13% (Fig. 4a). The population of leukocytes in colitis mice treated with PBS was significantly higher, composing more than 80% of the LP cells. While DNase-NZ could not completely inhibit the infiltration of immune cells, they were still able to significantly decrease its population (42.27 ± 3.22%) compared to PBS. Next, neutrophils were identified from leucocytes as cells were positive for both CD11c and Ly6G (Fig. 4b). Similar to the trend observed with leukocyte populations, DNase-NZ administration significantly decreased the proportion of neutrophils (25.41 ± 5.65%) compared to PBS (68.42 ± 5.47%). Interestingly, we also observed a significant decrease in leukocyte and neutrophil populations in mice treated with free DNase-I. To briefly investigate the possible mechanism related to these changes in neutrophil populations, we examined the gene expression level of *Cxcl5*, a chemokine involved in the recruitment of neutrophils [56] (Fig. 4c). The mRNA expression levels of *Cxcl5* were surprisingly suppressed in both mice treated with free DNase-I and DNase-NZ, indicating that downregulated neutrophil-recruiting chemokines may have caused low neutrophil populations. However, considering that free DNase-I treatment could not exhibit therapeutic effects in previous analyses (e.g., body weight change, MPO activity, histology), additional studies are required to fully interpret this phenomenon.

Finally, we further evaluated the mRNA expression of neutrophil elastase (*Elane*) and peptidyl arginine deiminase 4 (*Padi4*) from colon samples to examine whether or not the changes in neutrophil population were correlated to NETosis activity. *Elane* is a neutrophil-specific protease that promotes intracellular chromatin decondensation and prepares neutrophils before NETosis [57]. On the other hand, *Padi4* is an enzyme responsible for histone citrullination, an essential process in NETosis that initiates the formation of NETs [58]. These two enzymes work in tandem to produce NETs and are therefore direct indicators of NETosis. In colitis mice treated with DNase-NZ, both *Elane* (Fig. 4d) and *Padi4* (Fig. 4e) mRNA expression levels were comparable to those of control mice, indicating that NETosis activity was minimal despite the presence of infiltrated neutrophils (Fig. 4b). On the contrary, NETosis was highly activated in colitis mice treated with PBS, mesalamine, or free DNase-I, evidenced by the high expression levels of *Elane* and *Padi4*. Even though mice treated with free DNase-I exhibited decreased neutrophil populations, their NETosis activity was not altered, which is likely the reason why free DNase-I could not achieve therapeutic effects similar to those of DNase-NZ. Intriguingly, these results imply that the critical difference between free DNase-I and DNase-NZ was their ability to inhibit NETosis.

## Conclusions

Herein, we fabricated DNase-NZ to target NETs, utilizing DNase-I as a potential therapeutic material against IBD. DNase-NZ provided excellent stability to surface-immobilized DNase-I, while successfully preserving its enzymatic activity against NETs in vivo. When intra-rectally administered to DSS-induced colitis models, DNase-NZ treatment was able to significantly inhibit body weight loss, preserve colon length, reduce pro-inflammatory cytokine levels, and maintain the structural integrity of the colon. Moreover, our results demonstrated that DNase-NZ treatment could relieve the physiological symptoms of acute colitis and attenuate both neutrophil recruitment and NETosis in the colon. Both PLGA [59] and DNase-I [60] are approved by the Food and Drug Administration, making the current nanoplatform a highly biocompatible and safe approach in inhibiting NET-induced inflammations in IBD. Unlike other medications used for the treatment of IBD, DNase-I is also often well-tolerated with few side effects [61], making it a noteworthy IBD drug candidate. Nevertheless, there are also several limitations that should be addressed to further understand and potentiate the significance of DNase-NZ. Overproduced NETs are considered detrimental in IBD, but NETs, in general, also play important beneficial roles in immune defense [62]. Therefore, further studies are required to confirm whether prolonged DNase-NZ treatment affects the beneficial aspects of NETs as well. In addition, while oral administration represents a highly convenient approach for drug delivery in IBD, the current nanozyme platform is administered intrarectally. Hence, transitioning it to an oral platform holds the potential to significantly enhance its clinical relevance in the future.

### Supplementary Information

**Additional file 1: Figure S1.** Changes in daily body weights of normal and colitis mice (n = 5). Mice were provided 2.5% DSS dissolved in drinking water ad libitum for five days. From day 5 onward, the water supply was changed to normal drinking water until day 12. Data are presented as mean ± SEM.

**Additional file 2: Figure S2.** Unit measurements of DNase-NZ using the DNA degradation assay against various amounts of DNase-NZ (0.5, 1, 2.5, 5., 10, 12.5, 15, 25 μg). One unit was determined as 2.5 μg of DNase-NZ according to the absence of DNA.

**Additional file 3: Figure S3.** (a) Changes in daily body weight during treatment of various DNase-NZ doses against DSS-induced colitis in mice and (b) colon lengths measured after killing (n = 5). Mice were provided 2.5% DSS dissolved in drinking water ad libitum for five days. From day 5 onward, the water supply was changed to normal drinking water until day 8. PBS or DNase-NZ (50 U, 100 U, 200 U) was intra-rectally administrated daily for 7 days (Day 0–6). Data are presented as mean ± SEM. Statistical significance was assessed using a two-tailed Student’s t-test.

**Additional file 4: Figure S4.** (a) Minimum changes of body weight of mice during course of treatment. PBS, mesalamine (100 mg/kg), free DNase-I (500 U), or DNase-NZ (500 U) was intra-rectally administrated daily for 7 days (Day 0–6). (b) The spleen index was calculated as the ratio between the spleen and final body weight (Spleen weight in mg / Body weight in g) at day 10. Data are presented as mean ± SEM. Statistical significance was assessed using a two-tailed Student’s t-test. **p < 0.0021, ***p < 0.0002.

## Data Availability

All data generated or analysed during this study are included in this published article and its additional information files.
