# Supplementary figures and images for: Polymeric DNase-I nanozymes targeting neutrophil extracellular traps for the treatment of bowel inflammation
Source: Nano Converg. 2024 Feb 8;11:6. doi: 10.1186/s40580-024-00414-9 (PMC10853102; doi:10.1186/s40580-024-00414-9)

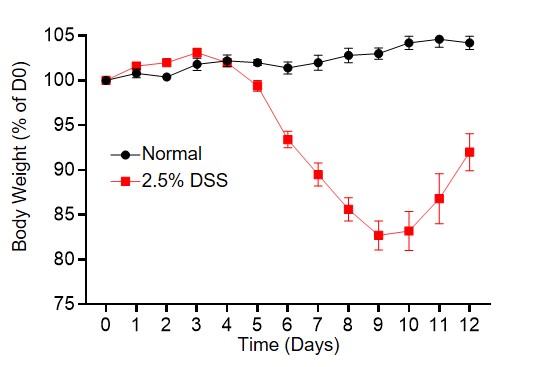

Supplement: Supplementary file 1 — Additional file 1: Figure S1. Changes in daily body weights of normal and colitis mice (n = 5). Mice were provided 2.5% DSS dissolved in drinking water ad libitum for five days. From day 5 onward, the water supply was changed to normal drinking water until day 12. Data are presented as mean ± SEM. [file 40580_2024_414_MOESM1_ESM.jpg]

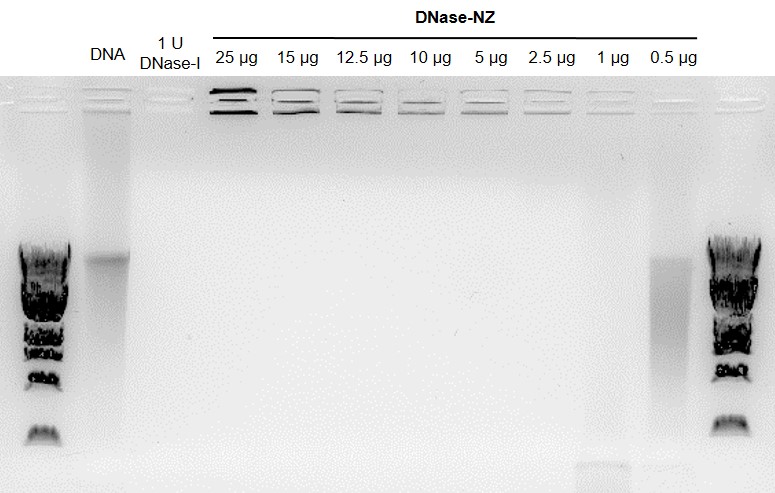

Supplement: Supplementary file 2 — Additional file 2: Figure S2. Unit measurements of DNase-NZ using the DNA degradation assay against various amounts of DNase-NZ (0.5, 1, 2.5, 5., 10, 12.5, 15, 25 μg). One unit was determined as 2.5 μg of DNase-NZ according to the absence of DNA. [file 40580_2024_414_MOESM2_ESM.jpg]

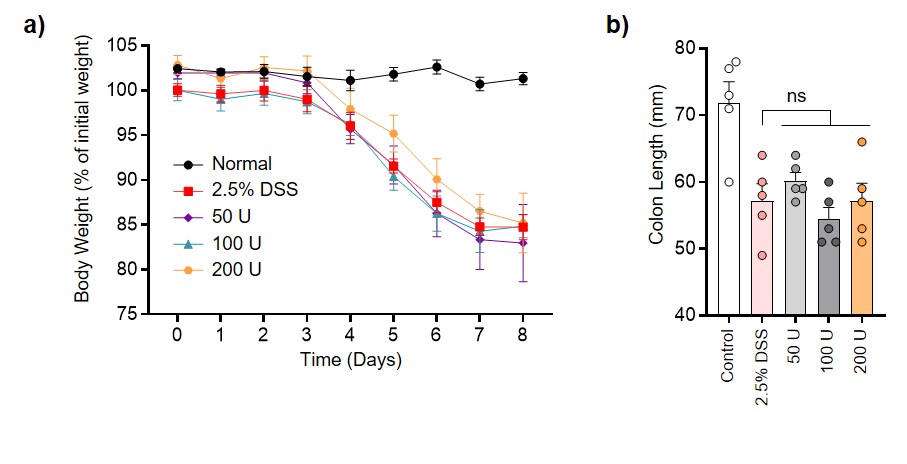

Supplement: Supplementary file 3 — Additional file 3: Figure S3. (a) Changes in daily body weight during treatment of various DNase-NZ doses against DSS-induced colitis in mice and (b) colon lengths measured after killing (n = 5). Mice were provided 2.5% DSS dissolved in drinking water ad libitum for five days. From day 5 onward, the water supply was changed to normal drinking water until day 8. PBS or DNase-NZ (50 U, 100 U, 200 U) was intra-rectally administrated daily for 7 days (Day 0–6). Data are presented as mean ± SEM. Statistical significance was assessed using a two-tailed Student’s t-test. [file 40580_2024_414_MOESM3_ESM.jpg]

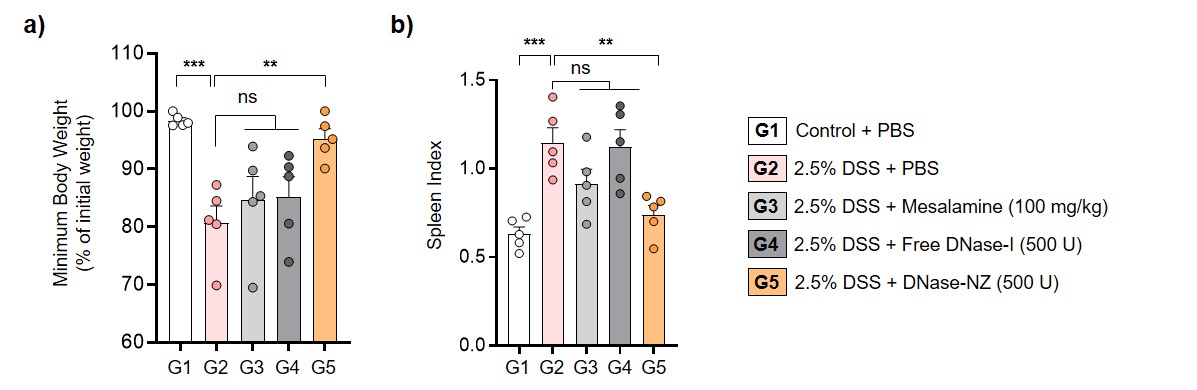

Supplement: Supplementary file 4 — Additional file 4: Figure S4. (a) Minimum changes of body weight of mice during course of treatment. PBS, mesalamine (100 mg/kg), free DNase-I (500 U), or DNase-NZ (500 U) was intra-rectally administrated daily for 7 days (Day 0–6). (b) The spleen index was calculated as the ratio between the spleen and final body weight (Spleen weight in mg / Body weight in g) at day 10. Data are presented as mean ± SEM. Statistical significance was assessed using a two-tailed Student’s t-test. **p < 0.0021, ***p < 0.0002. [file 40580_2024_414_MOESM4_ESM.jpg]
